# Knowledge mapping concerning applications of nanocomposite hydrogels for drug delivery: A bibliometric and visualized study (2003–2022)

**DOI:** 10.3389/fbioe.2022.1099616

**Published:** 2023-01-06

**Authors:** Hao Wang, Hongxun Fu, Yefan Fu, Lin Jiang, Liye Wang, Haibin Tong, Zuoxu Xie, Peng Huang, Meiyan Sun

**Affiliations:** ^1^ College of Laboratory Medicine, Jilin Medical University, Jilin, China; ^2^ Key Laboratory of Micro/Nano and Ultra-precision Manufacturing, School of Mechatronic Engineering, Changchun University of Technology, Changchun, China; ^3^ College of Pharmacy, University of Houston, Houston, TX, United States; ^4^ College of Life and Environmental Science, Wenzhou University, Wenzhou, China; ^5^ Department of Pharmacy, The Third Affiliated Hospital of Wenzhou Medical University, Wenzhou, China

**Keywords:** bibliometrics, knowledge mapping, nanocomposite hydrogels, drug delivery, citespace, bibliometrix

## Abstract

**Background:** Nanocomposite Hydrogels (NHs) are 3D molecular networks formed by physically or covalently crosslinking polymer with nanoparticles or nanostructures, which are particularly suitable for serving as carriers for drug delivery systems. Many articles pertaining to the applications of Nanocomposite Hydrogels for drug delivery have been published, however, the use of bibliometric and visualized analysis in this area remains unstudied. The purpose of this bibliometric study intended to comprehensively analyze the knowledge domain, research hotspots and frontiers associated with the applications of Nanocomposite Hydrogels for drug delivery.

**Methods:** We identified and retrieved the publications concerning the applications of NHs for drug delivery between 2003 and 2022 from Web of Science Core Collection Bibliometric and visualized analysis was utilized in this investigative study.

**Results:** 631 articles meeting the inclusion criteria were identified and retrieved from WoSCC. Among those, 2,233 authors worldwide contributed in the studies, accompanied by an average annual article increase of 24.67%. The articles were co-authored by 764 institutions from 52 countries/regions, and China published the most, followed by Iran and the United States. Five institutions published more than 40 papers, namely Univ Tabriz (*n* = 79), Tabriz Univ Med Sci (*n* = 70), Islamic Azad Univ (*n* = 49), Payame Noor Univ (*n* = 42) and Texas A&M Univ (*n* = 41). The articles were published in 198 journals, among which the International Journal of Biological Macromolecules (*n* = 53) published the most articles, followed by Carbohydrate Polymers (*n* = 24) and ACS Applied Materials and Interfaces (*n* = 22). The top three journals most locally cited were Carbohydrate Polymers, Biomaterials and Advanced materials. The most productive author was Namazi H (29 articles), followed by Bardajee G (15 articles) and Zhang J (11 articles) and the researchers who worked closely with other ones usually published more papers. “Doxorubicin,” “antibacterial” and “responsive hydrogels” represent the current research hotspots in this field and “cancer therapy” was a rising research topic in recent years. “(cancer) therapeutics” and “bioadhesive” represent the current research frontiers.

**Conclusion:** This bibliometric and visualized analysis offered an investigative study and comprehensive understanding of publications regarding the applications of Nanocomposite Hydrogels for drug delivery from 2003 to 2022. The outcome of this study would provide insights for researchers in the field of Nanocomposite Hydrogels applications for drug delivery.

## 1 Introduction

Hydrogel is a three-dimensional (3D) natural or synthetic polymer network composed of crosslinked hydrophilic chains (Gholamali and Yadollahi, 2020). The hydrophilic functional groups constituting the hydrogels make them have the superior ability to absorb and retain a plethora of water, biological fluid and solute molecules, swelling several times to tens of times the dry weight (Rafieian et al., 2019). Due to its swelling/de-swelling behavior, biocompatibility, environmental responsiveness and many other physical, chemical, electrical, and biological characteristics that mimic natural biological tissues, hydrogels have been applied in biomedical fields such as stem cell engineering, cancer research, drug delivery and tissue engineering (Taghizadeh et al., 2019; Liu et al., 2021; Jing et al., 2022). The 3D porous network of hydrogels is often used to retain, capture or release materials, which is particularly beneficial for drug delivery (Gaharwar et al., 2014; Taghizadeh et al., 2019). However, the medium mechanical properties and limited functionality of hydrogels prevent their further application in the biomedical field (Zhao et al., 2020). As a consequence, since nanocomposite hydrogels (NHs) were first demonstrated in 2002 (Haraguchi and Takehisa, 2002), they have captivated extensive attention and study from researchers due to the strategy to improve the performance of hydrogels by integrating the favorable characteristics of nanomaterials with 3D hydrogel networks (Zhao et al., 2020). NHs are 3D molecular networks formed by physically or covalently crosslinking polymer with nanoparticles or nanostructures (Merino et al., 2015; Zhao et al., 2020). A range of nanoparticles or nanostructures, including inorganic nanoparticles (such as clay, hydroxyapatite, carbon nanotubes, graphene, silica, silicates, and metal/metal-oxide nanoparticles) and organic/polymer nanoparticles (such as polymer nanoparticles, dendrimers, hyperbranched polyesters) are crosslinked with polymer networks to produce NHs (Sharma et al., 2017). The drug delivery systems are designed to keep, control and release the therapeutic agent, and their aim is to deliver the drug or any other active agent to the specific parts of the body with a predetermined release curve within a predetermined period of time, while avoiding possible toxicity, minimizing side effects, and increasing patients comfort by reducing the frequency of administration (Sirousazar, 2013; Xiao et al., 2019). NHs are undoubtedly suitable carriers for drug delivery systems. Because by developing nanocomposites with specific mechanical properties and functions, different types of NHs can 1)regulate the release behavior of therapeutic agents through their unique physical properties (e.g., swelling ratio, diffusion coefficient), 2)serve as therapeutic agent molecules library through the interactions between therapeutic agent molecules and nano-partials or hydrogel matrix, 3)be responsible for the multi-stimulus (i.e., external stimuli such as electric field, magnetic field, temperature and pH) response systems of drug delivery, 4)enhance the synergies (e.g., photothermal conversion effect) for disease treatment, achieve targeted drug delivery and so forth (Merino et al., 2015; Song et al., 2015; Li et al., 2021; Zhao et al., 2021).

In recent years, many excellent reviews have been made applications of nanocomposite hydrogels for drug delivery. However, researchers may miss many important facts due to the study of literature from one or several aspects, which is inevitable. For researchers, it is also a great challenge to explore the intrinsic relationship in a large number of literatures in related fields. As an advanced method of paper analysis bibliometrics focuses on bibliometric analysis in terms of countries, institutions, journals, authors, keywords and cited documents to determine the knowledge base, hotspots, frontiers and development trends of relevant scientific research fields (Chen, 2004; Ellegaard and Wallin, 2015; Gonzalez-Alcaide et al., 2017; Chen and Song, 2019). Information visualization based on computer science is one of the significant developments of bibliometrics, researchers can have a concrete and intuitive understanding of specific research fields through information networks and maps (Gonzalez-Alcaide et al., 2017). Bibliometrics has become a powerful tool for people to quickly grasp the scope and theme, history and current situation, frontiers and trends of a certain scientific research field, which has been widely used in many fields and achieved a lot of research results (Qi-Qi et al., 2016; Ke et al., 2020; Liu T. et al., 2021; Dong et al., 2022; Xiong et al., 2022). However, the bibliometric and visualized analysis for NHs is still lacking, especially their applications in drug delivery system.

In this study, we performed bibliometric analysis using bibliometrics software CiteSpace and the R-language package Bibliometrix to analyze the retrieved articles worldwide regarding the applications of NHs for drug delivery. We identified the knowledge domain, research hotspots in this research field, and shed light on the future directions based off our research outcomes.

## 2 Materials and methods

### 2.1 Data source and search strategy

Web of Science Core Collection (WoSCC) was searched to identify the English publications between 1 January 2002 (the first year NHs got reported), and 1 September 2022 (end date of the search) in the field of NHs application for drug delivery. WoSCC is a widely and commonly used database for bibliometric analysis. The query keywords included [TS= ((“nanocomposite hydrogel*”)) OR TS= (“hydrogel* nanocomposite*”))] AND TS= (“drug delivery”), with restriction to English literature. The qualified literature was selected for further bibliometric analysis, and additional references were identified through citation searches. After removing duplicates, titles and abstracts were screened for any mention of NHs applications for drug delivery by independent reviewers.

### 2.2 Statistical analysis

In this study, we used CiteSpace (version 6.1. R3), and the R-language package Bibliometrix 4.2.1 to conduct the bibliometric analysis. Data were extracted and analyzed by automatic algorithm and machine intelligence of bibliometrics software.

Bibliometrix is an R-tool for visualized networks and maps analysis of bibliometrics (Aria and Cuccurullo, 2017). Using Bibliometrix, we obtained the overall information, trend topics and landmark literature regarding the publications of the related research field, and conducted countries analysis, journals analysis, institutions analysis and authors productivity analysis.

CiteSpace is an analysis software of bibliometrics, which can be utilized to analyze the knowledge domain, research content, hot pots and frontiers of a certain specific research field by visualizing networks and maps (Chen, 2004; Chen and Leydesdorff, 2014). Using CiteSpace, we obtained the dual-map overlay of citations and conducted keywords analysis, references analysis, cluster analysis and analysis of collaboration among authors for the publications regarding the research field studied by this article. It should be pointed out here that clustering analysis has two evaluation indicators, namely modularity Q and mean silhouette value. When the values of these two indicators are greater than .3 and .5, respectively, the clustering results are considered to be meaningful and significant.

## 3 Results

### 3.1 Overall information of publications

**FIGURE 1:**
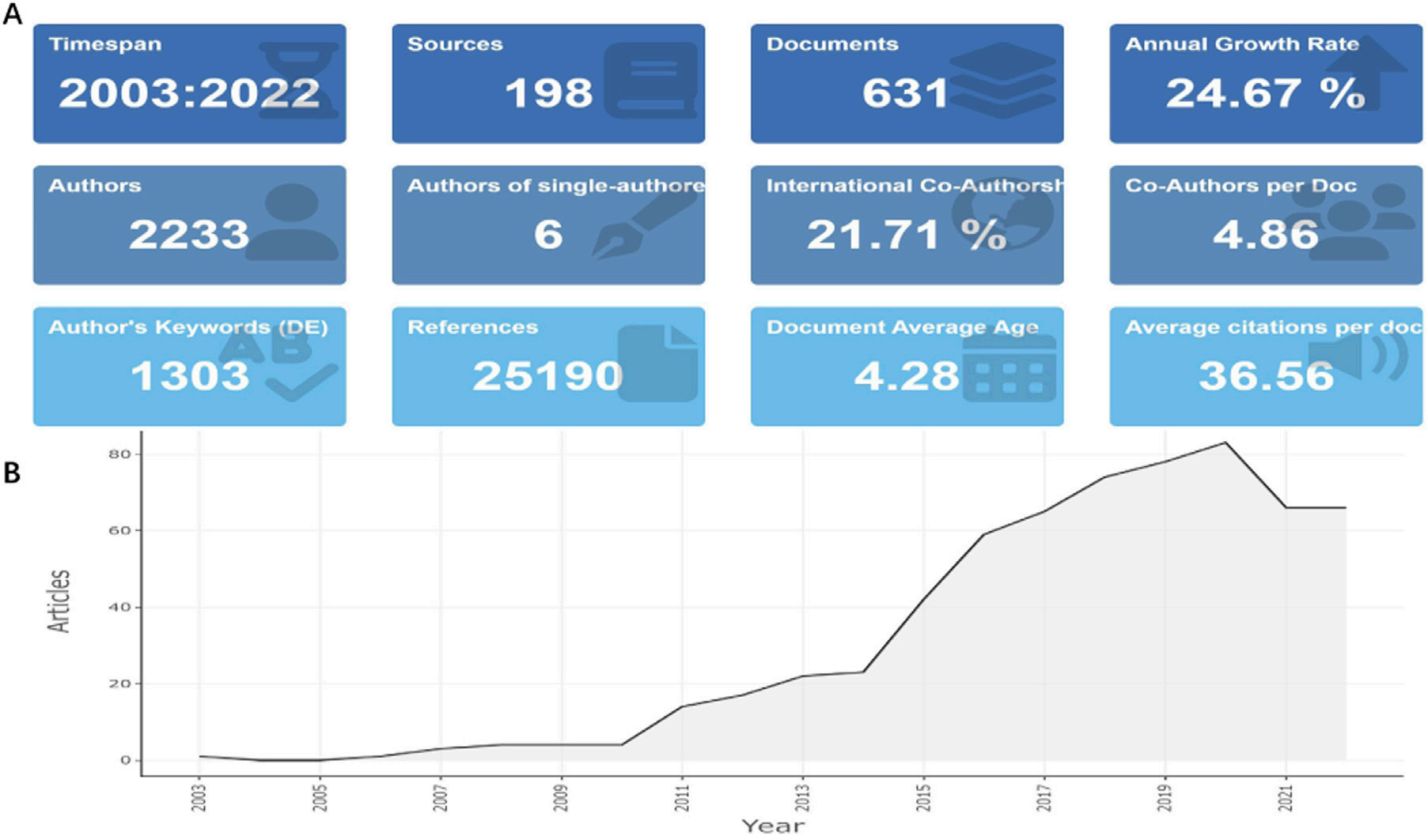
Main information **(A)** and annual scientific production **(B)** of publications concerning the applications of NHs for drug delivery.

We identified and retrieved 631 articles in total met the eligibility criteria from WoSCC. Figure 1A shows the study characteristics regarding the applications of NHs for drug delivery. Figure 1A demonstrates that 2,233 authors participated in the related studies worldwide, accompanied by an average annual article growth rate of 24.67% with 4.28 years as the average age, which confirmed that NHs application for drug delivery is a fairly new research field. As shown in Figure 1B, in the past 20 years, the number of annual publications in relevant fields has undergone a universal increase since 2003 (the first article was published). In particular, in the past decade (from 2013 to 2022), the number of publications has surged, accounting for 95.72% of the total. According to the annual scientific production map (Figure 1B), 82 publications were expected to be accomplished in 2022.

### 3.2 Analysis of countries/regions and institutions

**FIGURE 2:**
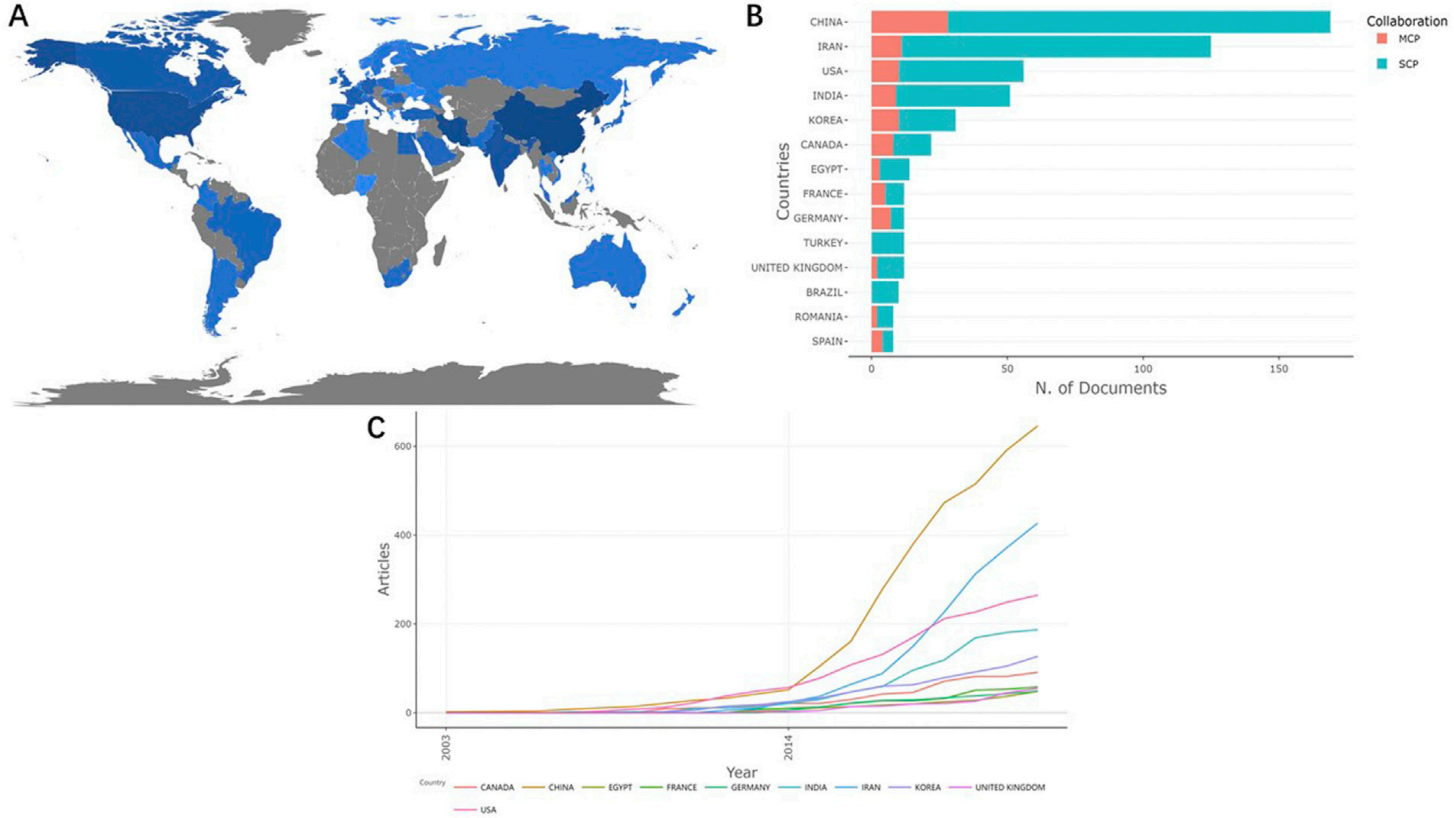
Contributions of different countries regarding the research of NHs applications for drug delivery. **(A)** Global country scientific production contributions (The depth of color represents the number of articles published); **(B)** Top 10 countries with the highest productivity (based on the countries where the corresponding authors come from); **(C)** Production of the top 10 countries with the highest productivity over time.

The articles we retrieved were co-authored by 764 institutions from 52 countries/regions. Figure 2A shows the global distribution of country scientific production regarding the field. The top 10 countries with the highest number of publications come from four continents, including four countries in Asia (China, Iran, India and South Korea), three in Europe (France, United Kingdom and Germany), two in North America (the United States and Canada) and one in Africa (Egypt). Figure 2B; Table 1 rank the publication number by the countries where the corresponding authors come from. Among the top 10 countries, China published the most, followed by Iran and the United States. In addition, since 2014, the number of publications on the applications of NHs for drug delivery in China has shown an increase over other countries (Figure 2C).

**TABLE 1:** Top 10 countries with the highest productivity of publications related to applications of NHs for drug delivery (based on the countries of the corresponding authors).

Country	Articles	SCP	MCP	Freq	MCP ratio
China	169	141	28	.268	.166
Iran	125	114	11	.198	.088
United States	56	46	10	.089	.179
India	51	42	9	.081	.176
Korea	31	21	10	.049	.323
Canada	22	14	8	.035	.364
Egypt	14	11	3	.022	.214
France	12	7	5	.019	.417
Germany	12	5	7	.019	.583
Turkey	12	12	0	.019	0
United Kingdom	12	10	2	.019	.167
Brazil	10	10	0	.016	0
Romania	8	6	2	.013	.25
Spain	8	4	4	.013	0.5

**FIGURE 3:**
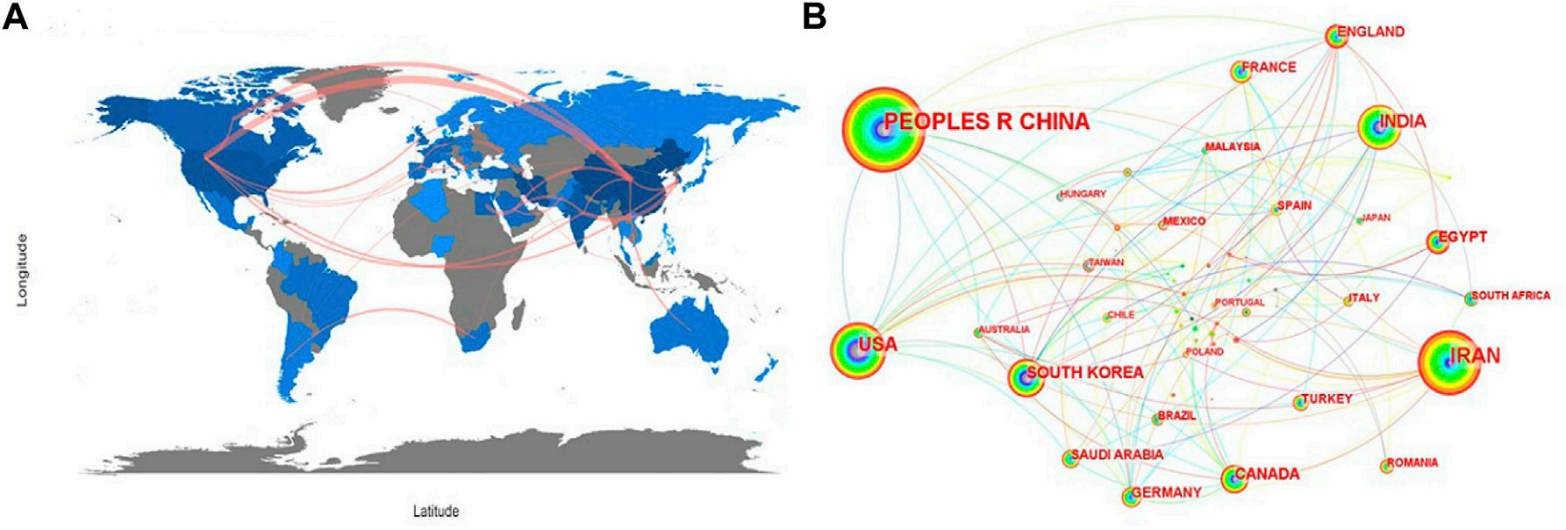
Cooperation of countries with regard to the applications of NHs for drug delivery. **(A)** The network map of cooperation relations between countries generated with R-Bibliometrix; **(B)** Visualized network map of cooperation relations between countries generated with CiteSpace.

The international collaboration relations were visualized in Figure 3A and Figure 3B. In these two network maps, different countries/regions have relatively close cooperation in the studies on the applications of NHs for drug delivery, among which China, Iran and the United States have larger nodes, indicating that these three countries had more cooperation with other countries. For example, China had close cooperation with the United States, South Korea, Japan, Australia, Germany, etc; Iran also had close cooperation with France, Poland, Canada, South Korea, etc.

**FIGURE 4:**
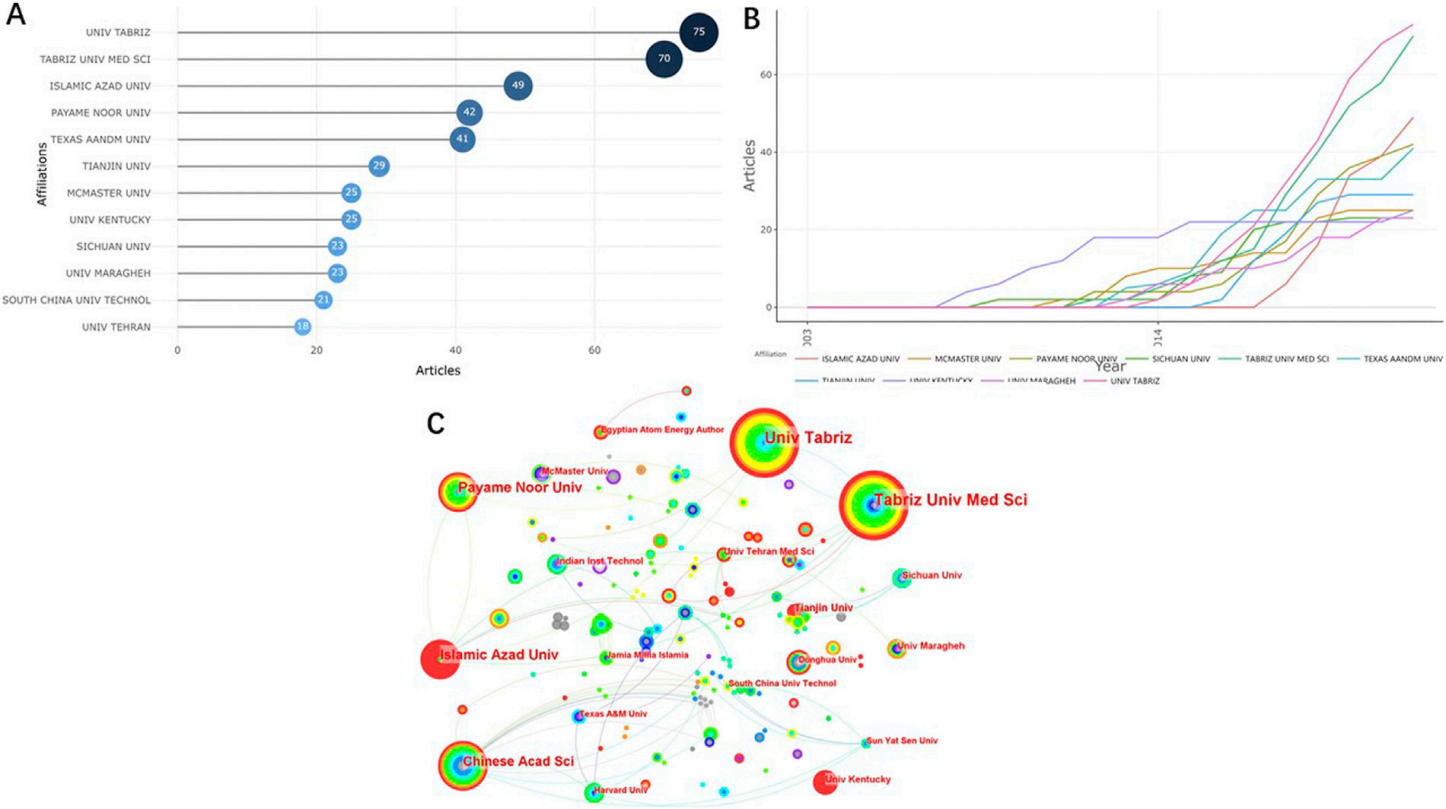
Visualized analysis of institutions concerning the publications of NHs applications for drug delivery. **(A)** The top 10 institutions with the most published papers; **(B)** Production of the top 10 institutions with the highest productivity over time. **(C)** The network map of cooperation relations between institutions.

The top 10 institutions with the highest number of publications in relevant research field are shown in Figure 4A. Half of the top 10 institutions distributing in four countries are located in Iran. Five institutions published more than 40 papers, namely Univ Tabriz (*n* = 79), Tabriz Univ Med Sci (*n* = 70), Islamic Azad Univ (*n* = 49), Payame Noor Univ (*n* = 42) and Texas A&M Univ (*n* = 41). The studies in the field we analysed did not start in Univ Tabriz until 2014, but the number of articles published by this university increased rapidly, and Univ Kentucky carried out this research earlier and continued to this day (Figure 4B). Figure 4C shows the collaboration relations between different institutions. As we can see, the four academic institutions from Iran had more cooperation, but most of them cooperated with institutions in their own countries. Research institutions in China and the United States, such as the Chinese Acad Sci and Harvard Univ, had more experience in cooperation with institutions in other countries.

### 3.3 Analysis of authors and journals

**FIGURE 5:**
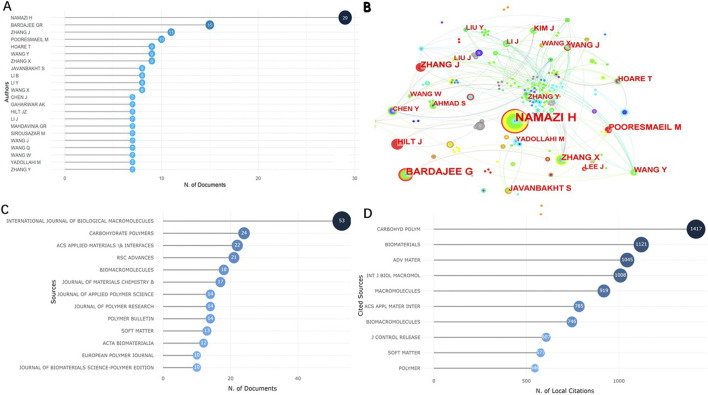
Visualized analysis of authors and journals concerning the publications of NHs applications for drug delivery. **(A)** The top 10 authors with the most published papers; **(B)** The network map of cooperative relations between authors. **(C)** The top 10 most productive journals; **(D)**The top 10 journals with the most local cited publications.

2,233 researchers co-authored the 631 literature, 22 of whom published more than six articles (Figure 5A). The most productive author was Namazi H (29 articles), followed by Bardajee G (15 articles) and Zhang J (11 articles). As shown in the authors’ cooperation network map (Figure 5B), researchers who worked closely with other ones usually published more papers.

The articles we analysed were published in 198 journals, among which International Journal of Biological Macromolecules (*n* = 53) published the most articles, followed by Carbohydrate Polymers (*n* = 24) and ACS Applied Materials and Interfaces (*n* = 22) (Figure 5C). The top 10 journals published 242 articles, accounting for 38.35% of the total. In addition, the map of most local cited sources was generated (Figure 5D), showing that the top three journals most locally cited were Carbohydrate Polymers, Biomaterials and Advanced materials.

**FIGURE 6:**
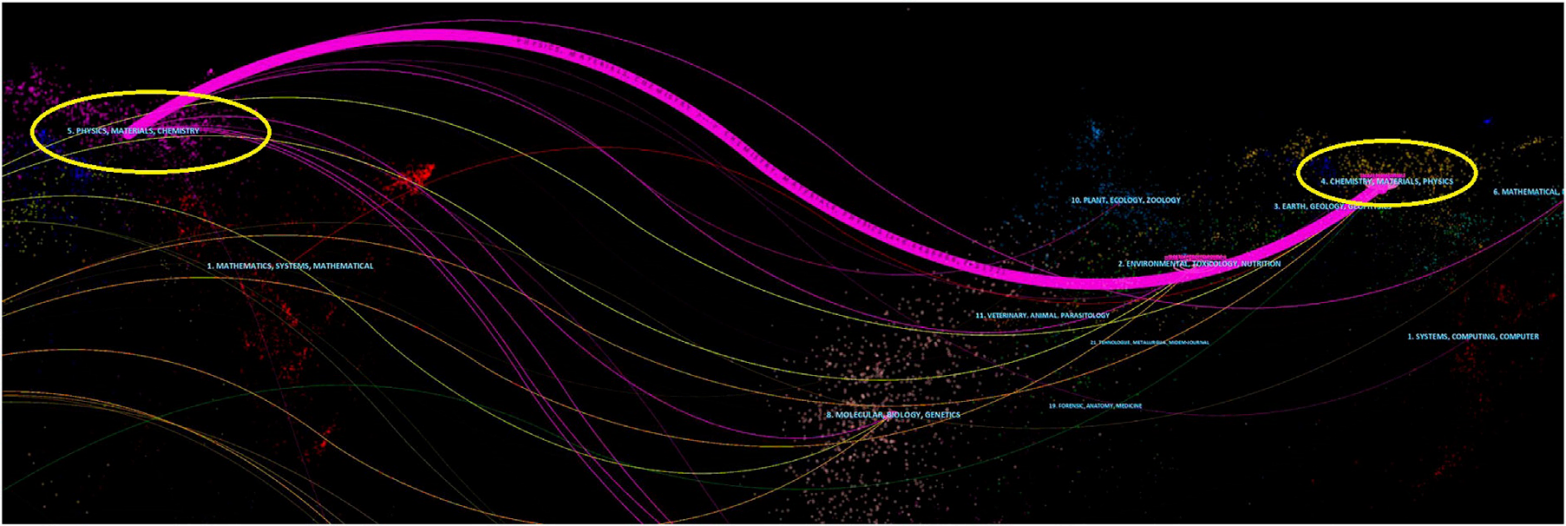
Part of dual-map overlay for journals related to the applications of NHs for drug delivery.

The dual-map generated by CiteSpace can indicate the topic distribution of journals through the main citation paths (Chen, 2017). The left and right sides of the dual-map overlay represent the citing journals and cited sources respectively, accompanied by the color stripes in the middle representing the citation paths. A main path and many minor paths were shown in the dual-map regarding the articles we retrieved. To more clearly describe the topic distribution of journals regarding the applications of NHs for drug delivery, we took a part of the dual-map (Figure 6). Publications concerning the applications of NHs for drug delivery were mainly published in Physics/Materials/Chemistry journals, while the cited literature was usually involved in Chemistry/Materials/Physics journals.

### 3.4 Keywords analysis

Keywords are the core content of academic literature, showing the research subjects or specific issues of the papers. The keywords with high frequency imply the hotspots in relevant fields, while the centrality of keywords indicates the importance and influence of the research content reflected by keywords in relevant research fields. Through keyword analysis, we could quickly understand the research scope, content and hotspots in specific fields (Qi-Qi et al., 2016).

**FIGURE 7:**
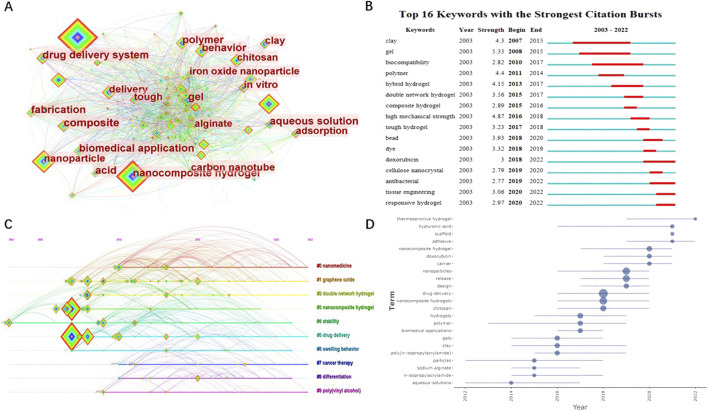
Visualized analysis of keywords regarding the publications on the applications of NHs for drug delivery. **(A)** The keywords co-occurrence network; **(B)** Keywords burst analysis indicated by the map of “Top 16 Keywords with the Strongest Citation Bursts”; **(C)** The timeline of clustering for keywords; **(D)** Map of keywords trend topics.

Table 2 shows the top 10 keywords in terms of frequency and centrality. The most central keyword was “polymer” (centrality: .12), indicating that it played an important role in the research field we analysed. Other important keywords included “nanoparticle” (frequency:144, centrality: .09) and “release” (frequency:121, centrality: .08). In addition, from the keywords co-occurrence network map (Figure 7A), we can know that, among the 1,302 keywords in the 631 articles, “carbon nanotubes,” “chitosan,” “clay,” “gel,” “iron oxide particles” and “*in vivo*” were all remarkable keywords except for some descriptive words.

**TABLE 2:** Some top keywords in terms of frequency and centrality.

Rank	Keywords	Count	Keywords	Centrality
1	drug delivery	379	polymer	.12
2	nanocomposite hydrogel	293	nanocomposite hydrogel	.09
3	nanoparticle	144	nanoparticle	.09
4	release	121	drug delivery system	.09
5	controlled release	70	release	.08
6	chitason	64	behavior	.08
7	behavior	60	delivery	.08
8	composite	59	acid	.08
9	graphene oxide	57	controlled release	.07
10	mechanical property	57	mechanical property	.07

Keywords can further represent the focus issues of relevant research fields in a certain period, and the recent burst keywords can represent the current research focuses (Qi-Qi et al., 2016; Zhong et al., 2020). As presented in Figure 7B, “clay” was once the most concerned hot spot in this research field, owing to its strongest citation bursts. Recent keywords with strong citation bursts were “doxorubicin,” “antibacterial” and “responsive hydrogels,” which could represent the current research hotspots in this field. Meanwhile, we could know that, although beyond the research content of this paper, the applications of NHs for tissue engineering may be another research hotspot at present, because “tissue engineering” was also a keyword with strong citation bursts.

Clustering analysis of keywords can be used to reveal the main topics of relevant research fields (Zhong et al., 2020). We used CiteSpace to obtain 10 clusters and generated a timeline of clustering (Figure 7C). The mean silhouette value of the cluster was .4321 and the modularity Q was .5453, indicating the clustering is reliable. As shown in Figure 7C, “nanomedicine” was the most important research area for the applications of NHS in drug delivery, and “cancer therapy” was a rising research topic in recent years. In addition, trends of keyword occurrences map (Figure 7D) shows that “thermosensitive hydrogel,” which belongs to the “responsive hydrogels” confirmed by map of burst keywords, was the latest research trend in this filed, indicating the potential research direction in the future.

### 3.5 References analysis

Cited and co-cited references are often used to analyse the domain and frontiers of knowledge in a certain field (Qi-Qi et al., 2016; Chen et al., 2010). According to the number of citations, we listed the top 10 of 25,151 local cited references related to this field (Table 3). A paper entitled “Hydrogels in drug delivery: Progress and challenges” published in the journal of Polymer ranked first having the most citation locally (cited 80 times) (Hoare and Kohane, 2008). This review discussed the *in vivo* delivery of hydrogels, the release kinetics of hydrogel drugs, and the nature of drugs delivered by hydrogels. The second and third places in Table 3 are “Nanocomposite Hydrogels: A Unique Organic–Inorganic Network Structure with Extraordinary Mechanical, Optical, and Swelling/De-swelling Properties” published in the journal of Advanced Materials (Haraguchi and Takehisa, 2002) and “Nanocomposite Hydrogels for Biomedical Applications” published in Biotechnology and Bioengineering (Gaharwar et al., 2014).

**TABLE 3:** Top 10 local cited references of publications related to the applications of NHs for drug delivery.

Rank	Cited references	Citations
1	Hoare and Kohane (2008), POLYMER, V49, P1993, DOI 10.1016/J.POLYMER. 2008.01.027	80
2	Haraguchi and Takehisa, (2002), ADV MATER, V14, P1120, DOI 10.1002/1,521–4,095 (20020816)14:163.0.CO; 2–9	76
3	Gaharwar et al. (2014), BIOTECHNOL BIOENG, V111, P441, DOI 10.1002/BIT.25160	65
4	Merino et al. (2015), ACS NANO, V9, P4686, DOI 10.1021/ACSNANO.5B01433	54
5	GONG JP, 2003, ADV MATER, V15, P1155, DOI 10.1002/ADMA.200304907	51
6	HOFFMAN AS, 2012, ADV DRUG DELIVER REV, V64, P18, DOI 10.1016/J.ADDR. 2012.09.010	48
7	PEPPAS NA, 2006, ADV MATER, V18, P1345, DOI 10.1002/ADMA.200501612	43
8	QIU Y, 2001, ADV DRUG DELIVER REV, V53, P321, DOI 10.1016/S0169-409X (01)00203–4	41
9	LEE KY, 2001, CHEM REV, V101, P1869, DOI 10.1021/CR000108X	40
9	Sun et al. (2012), NATURE, V489, P133, DOI 10.1038/NATURE11409	40
10	Haraguchi and Takehisa (2002), MACROMOLECULES, V35, P10162, DOI 10.1021/MA021301R	37
10	SCHEXNAILDER P, 2009, COLLOID POLYM SCI, V287, P1, DOI 10.1007/S00396-008-1949–0	37

**FIGURE 8:**
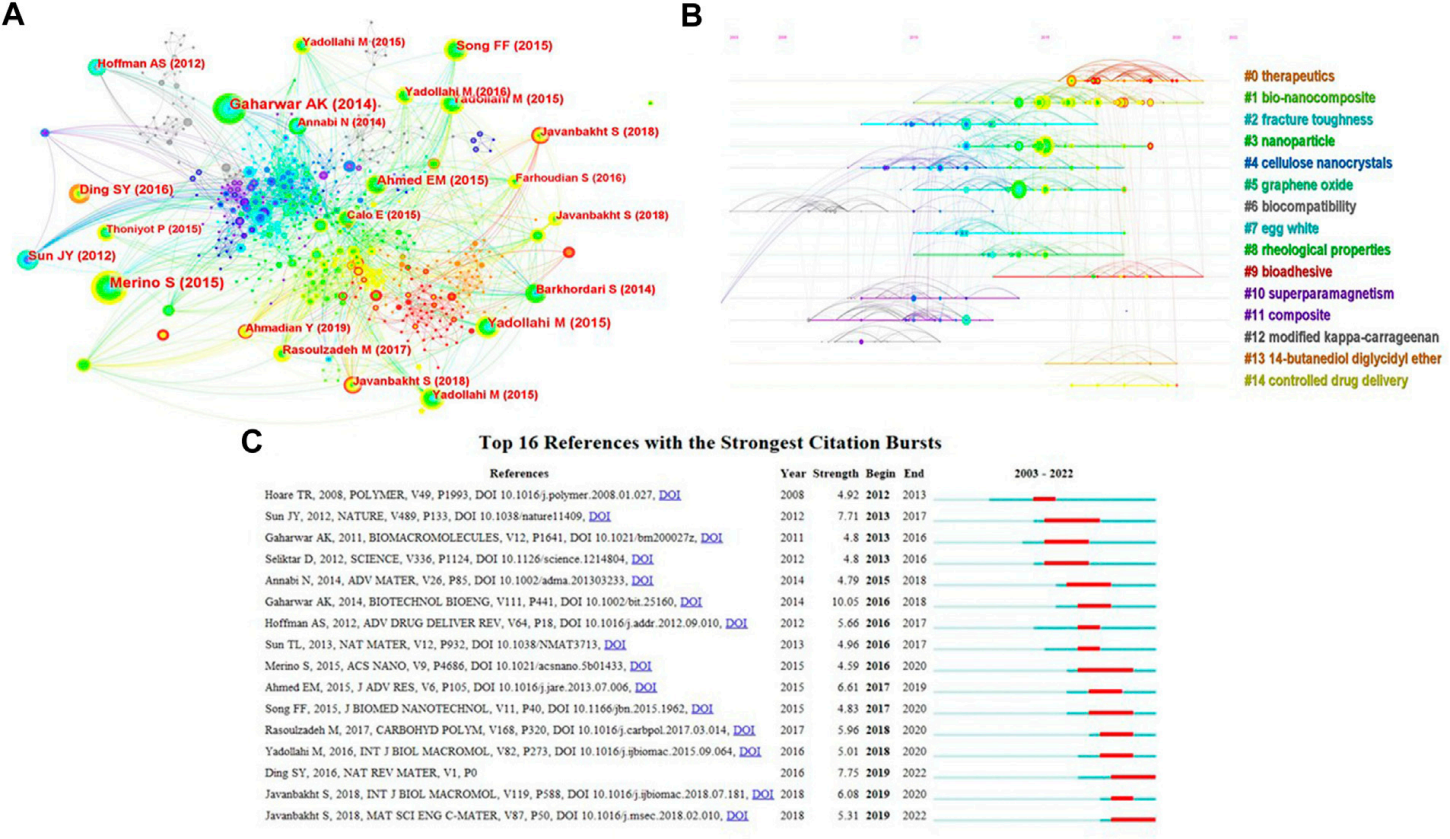
The analysis of references regarding the publications on the applications of NHs for drug delivery. **(A,B)** The visualized network map and clustering timeline of the co-cited references; **(C)** Top 16 References with the Strongest Citation Bursts.

The network map of co-cited references was generated by CiteSpace (Figure 8A). The top three co-cited references were (Merino et al., 2015), (Gaharwar et al., 2014) and (Yadollahi et al., 2015b), of which (Merino et al., 2015) and (Gaharwar et al., 2014) are review articles. The former focuses on discussing the synergies resulting from the combinations of NHs materials for on-demand drug delivery, and the latter focuses on the research progress and development trend of designing and developing NHs with customized physical properties and functions. (Yadollahi et al. (2015b) is a research article, demonstrating *in situ* synthesis, and characterization of antibacterial carboxymethyl cellulose (CMC)/CuO NHs. In addition, we obtained 15 clusters of cited references and visualized the clustering timeline (Figure 8B), which include “therapeutic,” “graphene oxide,” “bioadhesive,” “controlled drug delivery” and so forth, the modularity Q was .8474, and the mean silhouette value was .9183. We found that “fracture roughness,” “cellulose nanocrystals,” “rheological properties,” “superparamagnetism,” etc. are the fields that were studied earlier, while “therapeutics” and “bioadhesive” were the current research frontiers. Finally, we analysed the references with strong citation bursts, which are the foci of researchers in related fields for a period of time (Huang et al., 2019). As shown in Figure 8C a review by Gaharwar et al. (2014) had the strongest bursts, and this paper (Gaharwar et al., 2014) was also one of the most local cited, and co-cited literatures. The reference with the second strength was an article named “Highly stretchable and tough hydrogels” published in “Nature,” which reported the hydrogels synthesized from polymers with ionic and covalent crosslinking networks (Sun al., 2012). The two publications recently appeared in top 16 references with the strongest citation bursts were “Nanostructure-based plasmon enhanced Raman spectroscopy for surface analysis of materials” (Ding et al., 2016) and “Doxorubicin loaded carboxymethyl cellulose/graphene quantum dot nanocomposite hydrogel films as a potential anticancer drug delivery system” (Javanbakht and Namazi, 2018). They could represent the current research frontiers of NHs for drug delivery.

## 4 Discussion

**FIGURE 9:**
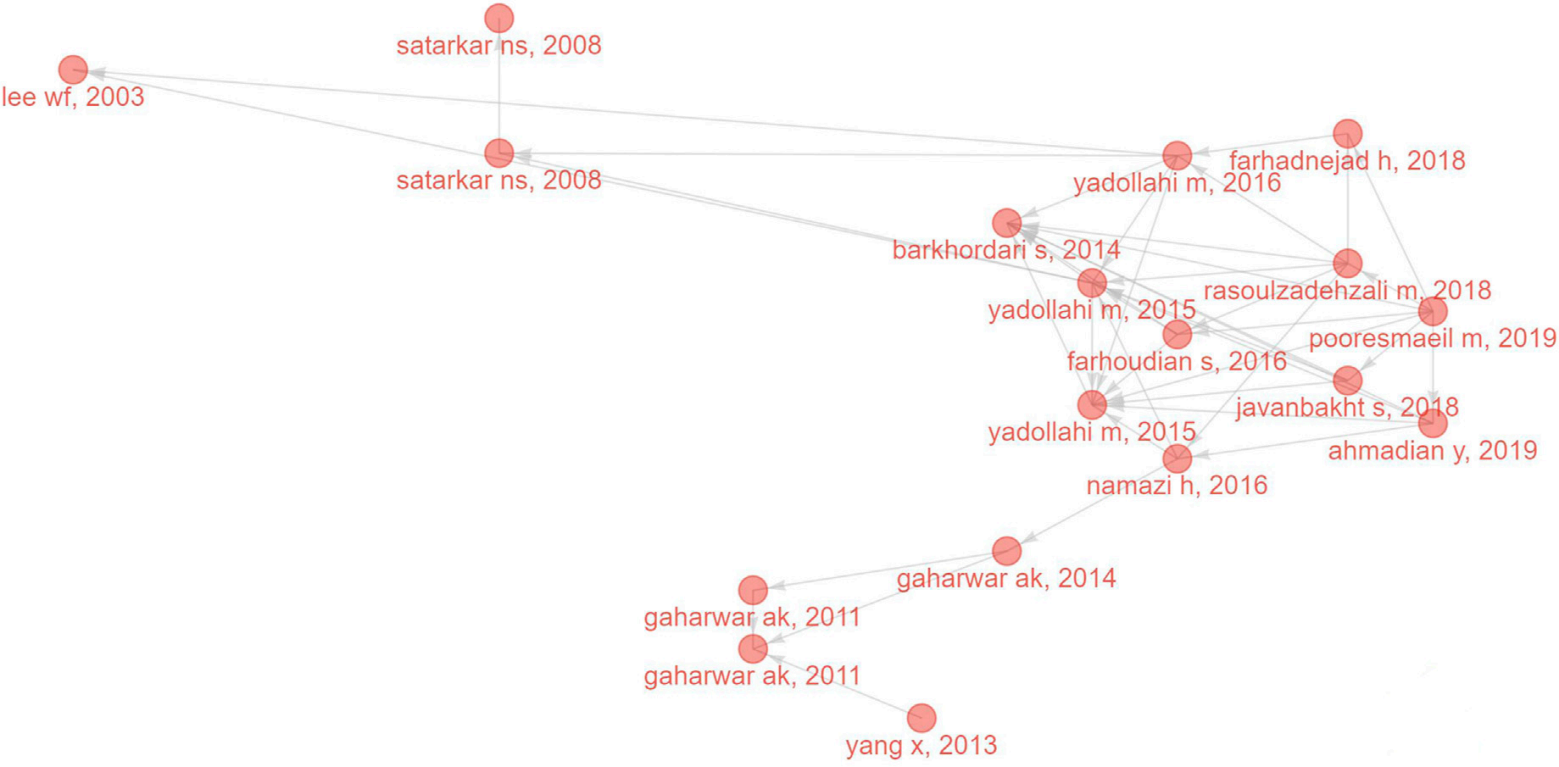
Landmark articles related to the applications of NHs for drug delivery.

In this study, we retrieved and performed analysis on 613 articles that scoped on the applications of NHs for drug delivery. With the help of bibliometric and visualized analysis, some milestone literature was also identified (Figure 9; Table 4). Since the publication of the first landmark article reporting the influence of N-isopropylacrylamide (NIPAAm)/organic montmorillonite (MMT) NHs on drug release behavior (Lee and Fu, 2003), the applications of NHs for drug delivery have attracted extensive attention from researchers. Subsequently, magnetic NHs with superparamagnetic Fe_3_O_4_ particles (Satarkar and Hilt, 2008; Satarkar and Zach Hilt, 2008), poly (ethylene glycol) (PEG)/hydroxyapatite nanoparticles (nHAp) NHs (Gaharwar et al., 2011a), poly (ethylene glycol) (PEG)/silicate nanoparticles NHs (Gaharwar et al., 2011b), injectable hydrogels reinforced with cellulose nanocrystals (CNCs) and aldehyde-functionalized CNCs (Yang et al., 2013), pH sensitive layered double hydroxides (LDH)/CMC NHs (Barkhordari et al., 2014), antibacterial chitosan/silver NHs (Yadollahi et al., 2015a), chitosan/ZnO NHs(Yadollahi et al., 2016), antibacterial chitosan/graphene oxide-Ag NHs (Rasoulzadehzali and Namazi, 2018) and other different types of NHs, were developed and investigated for drug delivery.

**TABLE 4:** Landmark papers concerning publications related to the applications of NHs for drug delivery.

Paper	Year	LCS	GCS
Lee and Fu., (2003), J APPL POLYM SCI DOI 10.1002/APP.12624	2003	10	141
Satarkar and Zach Hilt, (2008), ACTA BIOMATER DOI 10.1016/J.ACTBIO. 2007.07.009	2008	16	164
Satarkar and Hilt., (2008), J CONTROL RELEASE DOI 10.1016/J.JCONREL. 2008.06.008	2008	35	380
Gaharwar et al. (2011a), ACTA BIOMATER DOI 10.1016/J.ACTBIO. 2011.07.023	2011	12	159
Gaharwar et al. (2011b), BIOMACROMOLECULES DOI 10.1021/BM200027Z	2011	20	237
Yang et al. (2013), BIOMACROMOLECULES DOI 10.1021/BM401364Z	2013	10	199
Gaharwar et al. (2014), BIOTECHNOL BIOENG DOI 10.1002/BIT.25160	2014	65	682
Barkhordari et al. (2014), J POLYM RES DOI 10.1007/S10965-014-0454-Z	2014	29	110
Yadollahi et al. (2015a), INT J BIOL MACROMOL DOI 10.1016/J.IJBIOMAC. 2014.10.063	2015	23	100
Yadollahi et al. (2015b), INT J BIOL MACROMOL DOI 10.1016/J.IJBIOMAC. 2015.04.032	2015	28	151
Yadollahi et al. (2016), INT J BIOL MACROMOL DOI 10.1016/J.IJBIOMAC. 2015.09.064	2016	20	96
FARHOUDIAN S, 2016, INT J BIOL MACROMOL DOI 10.1016/J.IJBIOMAC. 2015.10.018	2016	15	77
NAMAZI H, 2016, INT J BIOL MACROMOL DOI 10.1016/J.IJBIOMAC. 2015.12.076	2016	16	115
FARHADNEJAD H, 2018, INT J BIOL MACROMOL DOI 10.1016/J.IJBIOMAC. 2018.01.061	2018	9	39
Rasoulzadehzali and Namazi (2018), INT J BIOL MACROMOL DOI 10.1016/J.IJBIOMAC. 2018.04.140	2018	13	65
Javanbakaht and Namazi (2018), INT J BIOL MACROMOL DOI 10.1016/J.IJBIOMAC. 2018.07.181	2018	16	89
Ahmadian et al. (2018), POLYM BULL DOI 10.1007/S00289-018-2,477–9	2019	16	56
Pooresmaeil et al. (2019), INT J BIOL MACROMOL DOI 10.1016/J.IJBIOMAC. 2019.08.060	2019	9	53

In recent years, synthesis and drug delivery application of PVA/CuO NHs (Ahmadian et al., 2018), as well as the nanohybrid (5-fluorouracil loaded in LDH(Zn/Al)) encapsulated with CMC (Pooresmaeil et al., 2019), which was developed for oral delivery of colon cancer drugs, were landmark research results. In general, as a fairly new research field, the research outcome related to the applications of NHs for drug delivery have shown an overall upward trend. Especially in the past 10 years, according to the proportion of publications, we noticed that there has been an explosion of activity in the field.

In terms of the number of publications related to the applications of NHs for drug delivery, China was the country published the most in this area. Tianjin Univ from China was one of the top 10 institutions having the most publications, and Zhang J from this institution ranked third among the most prolific authors. The research team of Tianjin Univ has been committed to the research of NHs applied to drug delivery for a long time. A recent paper of this institution demonstrated the target drug delivery NHs which were selectively delivered to the tumour site (Liu et al., 2021a). In addition, Iran was an important research force in this field, not only because it ranked second in terms of the number of publications, but also because Namazi H, the most prolific author, comes from Iran’s Univ Tabriz. Namazi’s research team recently reported a nanocomposite hydrogel based on hyperbranched polymers for cancer treatment (Aslani and Hassan, 2022). There were four developing countries (China, Iran, India and Egypt) among the top 10 countries with the largest number of publications, which indicates that developing countries contribute in the applications of NHs for drug delivery. Nevertheless, Iran and its research institutions have not cooperated closely with major developed countries in Europe and the United States in this field. From the bibliometric analysis above, we found that academic cooperation is often conducive to the output of research results, whether for countries or institutions and individuals. Therefore, serval research institutions and individuals could further cooperate closely to move the development of this research field forward.

The journal of International Journal of Biological Macromolecules had the most publications in the relevant field so far, indicating the journal made outstanding contributions to the research of NHs applications for drug delivery and was popular with researchers. For example, the journal recently reported a camellia sinensis-loaded nanocomposite hydrogel as a wound dressing (Hezari et al., 2022). Through comprehensive analysis of published and cited journals, we found that the topics in this research field were mainly distributed in chemistry, materials science, physics, and biomedicine, indicating that the applications of NHs for drug delivery is an interdisciplinary research field. This is also supported by the main paths shown on the dual-map.

The keyword with the highest centrality was “polymer,” in fact, hydrogels are polymers in nature. In addition, polymerization or cross-linking between nano-partials and hydrogels, and polymer-based nanoparticles are also important contents of research in this field (Gholamali and Yadollahi, 2020). Some other prominent keywords, such as “carbon nanotubes,” “chitosan,” “clay,” “iron oxide particles,” are also the main sources of nanoparticles and nanostructures constituting NHs (Merino et al., 2015; Song et al., 2015; Gholamali and Yadollahi, 2020). For instance, Jeddi and Mahkam prepared CMC-alginate/chitosan nanocomposite hydrogel magnetized by Fe3O4 nanoparticles for pH sensitive drug release (Karzar Jeddi and Mahkam, 2019). One of the recent keywords with strong citation bursts was “doxorubicin” which is an anti-tumor drug, indicating cancer therapy is a current research hot spot for NHs for drug delivery. This coincided with the result of keywords clustering timeline analysis, which showed that cancer treatment was a rising research topic in recent years. Gholamali et al. (2021) reported CMC/Starch/ZnO NHs used as carriers of doxorubicin for controlled delivery of anticancer drugs (Gholamali and Yadollahi, 2020). Some other NHs for anticancer drug delivery and cancer treatment have also been widely developed (Chen et al., 2012; Li et al., 2016; Taleblou et al., 2020; Gao et al., 2021). Another keyword appeared recently in TOP 16 keywords with the strongest citation bursts was “responsive hydrogels”, which includes “thermosensitive hydrogel” generated by trends of keyword occurrences map, indicating that responsive hydrogels are the current research hotspots and direction. Various stimuli responsive NHs have been investigated for controlled drug delivery, such as pH responsive (Barkhordari et al., 2014), electro-responsive (Yan et al., 2012), thermosensitive (Zhang et al., 2021), magnetic responsive (Satarkar and Hilt, 2008) and light-responsive (Yan et al., 2012) ones. Moreover, targeted drug delivery has received more attention, especially in cancer treatment. Targeting drug delivery requires the ligands recognize and react to specific receptors. The nano-parts of NHs are conducive to the conjugation of active targeting agents, which provides great potential for targeted drug delivery. A paper reported that targeting ligands could easily attach to the active functional groups of CMCs (Upadhyaya et al., 2014), which attracted the attention of researchers. It should be pointed out here that the responsive NHs are not limited to a single response. For example, thermal and pH responsive NHs with surface conjugated nanoparticles have been synthesized, which are further functionalized by tumor targeting peptides (Su al., 2013).

Notably, two of the top three most local cited references were reviews, which focused on the key issues in the clinical applications of NHs regarding drug delivery, and the application potential of NHs for biomedical field (Hoare and Kohane, 2008; Gaharwar et al., 2014). Another article in the top three groundbreaking reported the synthesis and characterization of NHs, laying a foundation for their applications in biomedical field (Haraguchi and Takehisa, 2002). The local cited references reflect the domain of knowledge in this research field to a great extent. One of the top three references most co-cited reported the synthesis and characterization of an antibacterial nanocomposite hydrogel (Yadollahi et al., 2015b). “Antibacterial” happened to be a recent keyword with strong citation bursts, which also indicates to a certain extent that the study of antibacterial NHs is one of the current focuses in this field. For example, a recent paper demonstrated a nanocomposite hydrogel with antibacterial properties used for bone defect repair (Li et al., 2022).

We noticed that from the timeline of references clustering that, “therapeutics” is a Frontier in the research field we analysed in recent years, which also corresponds to the fact that the “cancer therapy” we identified above is a current research hot spot in this field. Similarly, “bioadhesive” is another research Frontier in this field. Bioadhesive NHs can adhere to biological tissues for an extended time, which is very important for prolonging drug delivery and effective dosage applications, such as oral, rectal, nasal, and vaginal administration. Tan et al. (2019) reported a bioadhesive nanocomposite hydrogel functionalized by (3aminomethylphenyl) boronic acid-conjugated chondroitin sulfate (APBA-CHS), which can improve the delivery of drugs and has a good prospect for the treatment of eye diseases such as dry eye syndrome. One of publications recently appeared in top 16 references with the strongest citation bursts was an article stating nanocomposite hydrogels for cancer treatment (Javanbakht and Namazi, 2018), which is consistent with the research hotspot and Frontier of NHs for drug delivery that we have previously identified. Another paper with high citation bursts recently appeared was a review discussing Raman spectroscopy enhanced by nanostructure-based plasmon (Ding et al., 2016). Enhanced Raman spectroscopy can analyse the composition of nanoscale mixtures (Ding et al., 2016), which can be used to identify the structural compositions and nanoparticle distribution of NHs (M.Kurdtabar et al., 2021). Meanwhile, metal nanoparticles-based NHs can also be used as substrates for surface enhanced Raman spectroscopy (Alvarez-Puebla et al., 2009; Vianna et al., 2016). More importantly, methods based on surface enhanced Raman spectroscopy have been widely used in the detection of biomarkers, especially for the detection of precancerous and malignant tissues (Allayla et al., 2021). Given that cancer treatment is a hot spot and Frontier in the field of NHs applications for drug delivery, the citation of this review increased within a short time period.

The limitations of this study exist as follows: First, we retrieved the articles reporting the applications of NHs for drug delivery merely from the database WoSCC. Second, even for articles included in the WoSCC database, our search excluded the new publications after 1 September 2022 (the search end date). Besides, machine algorithms were utilized for analysis in this study, which would inevitably lead to insufficient human intervention.

## 5 Conclusion

This bibliometric analysis identifies the investigative studies that are associated with the applications of NHs for drug delivery 2003–2022, using the bibliometric analysis tools CiteSpace and R-bibliometrix. NHs for drug delivery have been widely studied as a booming interdisciplinary research field. Based off our analyses, we found that developing countries, including China and Iran, contribute the most in this field, and International Journal of Biological Macromolecules published the most in this field. Namazi H, Bardajee G and Zhang J had published a lot of papers in this research area. Academic collaboration among countries, institutions and individuals promotes the rapid development of the field. Cancer therapy, responsive hydrogels and antibacterial NHs are currently research hotspots, and the study of anticancer drug delivery and bioadhesive NHs is at the forefront of this field. In addition, thermosensitive hydrogel, as a kind of responsive hydrogels, represents a potential research direction at present. In a word, this paper will provide researchers in related fields with profound insights and a novel method of literature analysis.

## Data Availability

The original contributions presented in the study are included in the article/Supplementary Material, further inquiries can be directed to the corresponding authors.
